# Chronic Beryllium Disease and Sensitization at a Beryllium Processing Facility

**DOI:** 10.1289/ehp.7845

**Published:** 2005-05-26

**Authors:** Kenneth Rosenman, Vicki Hertzberg, Carol Rice, Mary Jo Reilly, Judith Aronchick, John E. Parker, Jackie Regovich, Milton Rossman

**Affiliations:** 1 Michigan State University, East Lansing, Michigan, USA; 2 Emory University, Atlanta, Georgia, USA; 3 University of Cincinnati, Cincinnati, Ohio, USA; 4 University of Pennsylvania, Philadelphia, Pennsylvania, USA; 5 West Virginia University, Morgantown, West Virginia, USA

**Keywords:** beryllium, chronic beryllium disease, epidemiology, exposure–response, lymphocyte proliferation testing

## Abstract

We conducted a medical screening for beryllium disease of 577 former workers from a beryllium processing facility. The screening included a medical and work history questionnaire, a chest radiograph, and blood lymphocyte proliferation testing for beryllium. A task exposure and a job exposure matrix were constructed to examine the association between exposure to beryllium and the development of beryllium disease. More than 90% of the cohort completed the questionnaire, and 74% completed the blood and radiograph component of the screening. Forty-four (7.6%) individuals had definite or probable chronic beryllium disease (CBD), and another 40 (7.0%) were sensitized to beryllium. The prevalence of CBD and sensitization in our cohort was greater than the prevalence reported in studies of other beryllium-exposed cohorts. Various exposure measures evaluated included duration; first decade worked; last decade worked; cumulative, mean, and highest job; and highest task exposure to beryllium (to both soluble and nonsoluble forms). Soluble cumulative and mean exposure levels were lower in individuals with CBD. Sensitized individuals had shorter duration of exposure, began work later, last worked longer ago, and had lower cumulative and peak exposures and lower nonsoluble cumulative and mean exposures. A possible explanation for the exposure–response findings of our study may be an interaction between genetic predisposition and a decreased permanence of soluble beryllium in the body. Both CBD and sensitization occurred in former workers whose mean daily working lifetime average exposures were lower than the current allowable Occupational Safety and Health Administration workplace air level of 2 μg/m^3^ and the Department of Energy guideline of 0.2 μg/m^3^.

Researchers early on recognized that chronic beryllium disease (CBD) occurred after both high and low levels of exposure and hypothesized that the disease was immunologically mediated (Sterner and Eisenbud 1951). Subsequent work has confirmed the importance of cellular immunity to beryllium in the pathogenesis of CBD (Rossman 2001). The factors that determine why some individuals develop cellular immunity to beryllium while others do not still need to be elucidated. Medical screenings of beryllium-exposed workers consistently demonstrate that a larger percentage of individuals will have a positive blood lymphocyte proliferation test to beryllium (become sensitized) than will be diagnosed with CBD (sensitization and granuloma in lung parenchyma) (Henneberger et al. 2001; Kelleher et al. 2001; Kreiss et al. 1993a; Stange et al. 2001). It is not known what proportion of individuals who are sensitized to beryllium will progress to develop CBD. Furthermore, there is a varied clinical presentation of patients with CBD and variability in its progression (Newman et al. 1996; Rossman et al. 1999).

The current occupational air standard for beryllium, first proposed in 1951, was based on the toxicity of other metals such as arsenic, lead, and mercury and modified to reflect beryllium’s lower atomic weight and concern about its greater toxicity (Eisenbud 1982). Epidemiologic health outcome and exposure studies were not used to develop the initial time-weighted average permissible exposure level of 2 μg/m^3^. Fifty years later, this remains the current air level that Occupational Safety and Health Administration (OSHA) enforces in the workplace.

Recent studies looking at beryllium disease and exposure have either used a surrogate of exposure (i.e., months of exposure, percent exposed to unfired beryllium oxide) or calculated exposure metrics and found increased disease with some parameters of increased exposure (Henneberger et al. 2001; Kelleher et al. 2001; Kreiss et al. 1993b, 1997; Viet et al. 2000). One study found an exposure–response relationship for sensitization with CBD but not for sensitization without CBD (Viet et al. 2000). Other work has addressed the possibility of particle size (McCawley et al. 2001), skin absorption (Tinkel et al. 2003), and/or genetic susceptibility (Saltini et al. 2001) as important factors that confound a straightforward exposure–response relationship.

We investigated possible exposure–response relationships separately for various measures of exposure, including mean, peak, and cumulative metrics and differing chemical and physical forms for the development of beryllium sensitization and for the development of CBD.

We have also assessed whether the current OSHA (2005) and Department of Energy (DOE 1999) permissible levels were protective against the development of CBD and sensitization.

## Materials and Methods

The cohort was composed of workers from a beryllium production facility in eastern Pennsylvania, which operated from 1957 to 1978. The names of former workers with at least 2 days of work up to 31 December 1969 who had previously been identified from personnel records and matched with Social Security Administration Form 941 records by the National Institute for Occupational Safety and Health (NIOSH), as part of a seven-company mortality study, were obtained from NIOSH (Ward et al. 1992). The last owner of the facility provided the names of workers, social security numbers, demographic information, and the last known address of all individuals who began work from 1 January 1970 until the plant closed in 1978.

Because this study was a cooperative effort with NIOSH, addresses from the last income tax filing of members of the cohort were obtained by NIOSH from the Internal Revenue Service. NIOSH had previously ascertained the vital status of the cohort as of 31 December 1988 using the Social Security Administration, the Internal Revenue Service, post office cards mailed to the last known address, the Department of Veterans Affairs, the Health Care Finance Administration, and the National Death Index (Ward et al. 1992).

We mailed the initial invitation to participate in the medical screening program to the last known address of all members of the cohort not known to be deceased as of 31 December 1988. The mailing included a cover letter about the study, a fact sheet about beryllium, a one-page two-sided questionnaire, and a postage-paid envelope. The questionnaire requested demographic information and had questions about previous lung disease, smoking history, and work history at the beryllium facility.

We attempted to contact everyone who did not return the questionnaire. This included multiple phone contacts or actual visits to the person’s home if telephone contact was unsuccessful. Internet address searches using search engines such as Yahoo! and Netscape were performed to locate current mailing addresses of individuals with returned mailings. In addition, we used the Social Security Death Index (Ancestry.com 2005) to help determine vital status of individuals. Local staff in the two communities not only made visits to last known addresses but also asked the long-term workers to assist in identifying individuals who could not be located.

All individuals located, whether or not they participated in the medical screening or completed a questionnaire, received a subsequent mailing summarizing the results of the screening and notification of federal legislation passed in the fall of 2000 that provided compensation for workers with CBD and coverage for medical costs for follow-up of workers with beryllium sensitization from this facility.

All individuals located had the opportunity to have a blood lymphocyte proliferation test for beryllium (BeLPT), a posterior–anterior chest radiograph, and simple spirometry. Before the testing, we obtained consent to conduct testing from the individual. In addition, each participant completed a questionnaire on other work exposures that might contribute to respiratory deficiencies. This included other possible sources of beryllium exposure as well as exposure to asbestos, coal, and silica.

Medical testing was performed at two primary sites in the community in eastern Pennsylvania. For individuals who had moved to other parts of the country, medical testing was performed in a location convenient to the individual (i.e., personal physician, local medical centers, etc.).

Blood was collected Monday through Thursday and shipped for next morning delivery. All blood was processed the next day and analyzed. All BeLPT was performed at the University of Pennsylvania. Any individual with a positive BeLPT test was offered a repeat test. If an individual’s results were negative on the repeat test, then the individual was offered the opportunity to repeat the blood test 1 year later.

A panel of three “B” readers interpreted all chest radiographs. One B reader was a radiologist (J.A.), one a pulmonologist (J.E.P.), and one an internist and occupational medicine physician (K.R.). At least two B readers had to classify a radiograph with ≥1/0 profusion in order for a radiograph to be classified as positive for parenchymal disease.

Any individual who had two positive BeLPTs and/or a consensus chest radiograph reading of ≥1/0 for profusion was referred to the University of Pennsylvania for follow-up testing, which consisted of a posterior–anterior chest radiograph, a BeLPT, an electrocardiogram, a complete medical history including respiratory symptoms using a standardized collection instrument, and bronchoscopy with both bronchial biopsy and lymphocyte testing of lavage fluid for beryllium. All bronchoscopies were performed by a single pulmonologist (M.R.).

Whether or not an individual had CBD or beryllium sensitization was decided by consensus by the internist/occupational physician (K.R.) and pulmonologist (M.R.). Table 1 outlines the criteria used to categorize the medical testing results. However, only individuals who had bronchoscopy were used in the analysis describing the predictive power of radiographs or BeLPT.

All individuals received a letter with the results of their initial screening and, where applicable, a letter with the results of the follow-up testing. The Human Subject Review Boards of Emory University, Michigan State University, the University of Cincinnati, and the University of Pennsylvania approved this study.

Through discussions with long-term production and management employees, we identified major changes in the process and engineering/work practice controls. Trends in the exposures over time were evaluated in relation to dates of process changes and visually from plots of the data to identify other time points at which exposure measurements indicated a change in conditions.

Exposure had been monitored at the facility using a method that combined the concentration at each task performed by a worker, weighted by the duration in the shift of that task; the products of concentration and duration at all tasks performed as part of a job were summed and divided by the duration to the shift. This final value was called the daily weighted average (DWA) exposure. Data accumulated over the operating history of the plant were identified and computerized. Using this information, a task exposure matrix (TEM) and a job exposure matrix (JEM) were constructed (Chen 2001). Task-related exposure measurements were available for two time periods, 1957–1962 and 1971–1976. Because the data most closely followed a log-normal distribution, the geometric mean was calculated for each task-year combination. For years with no measurements, we estimated exposures by interpolating between the previous and subsequent values. For example, if measurements were available for 1957, 1958, and 1959 but not for 1960, the 1959 value was entered into the TEM. The plant history was used to develop a strategy for imputing values from 1963 to 1971. We used the mean of task estimates for 1962 and 1971 for the period 1963–1969; because of the engineering changes in 1970, the 1971 values were used for 1970. Estimates for 1976 were used for the remaining years of plant operation, based on employee interviews. For tasks never measured, the task in the same work area most similar to the unmeasured task was identified with the assistance of long-term employees; the exposure value for the measured task was entered into the TEM for the unmeasured task.

We completed the JEM by first calculating the geometric mean exposure for each year in which at least one DWA measurement was available. Exposure estimates for job–year combinations without measurements were estimated based on the plant history of engineering changes. In the absence of information showing production or control technology changes in years before or after measurement data, the measurements were assumed valid and extended to the empty cells in the JEM. Where increases or decreases in exposure were justified from the plant history, we used analysis of variance (ANOVA) to evaluate the significance of the change in exposure. Where statistically significant changes were identified, the new value was entered into the cell of the JEM.

For 39 of the 130 job titles, no measurements were available for the job in any year. For each of these jobs, we used information from the long-term workers to identify the job with tasks most similar to it with measurements. The time–activity pattern needed for the evaluation of exposure was developed and used to calculate a DWA estimate of exposure using data in the TEM.

The values were reviewed by a group of long-term employees who represented experience in all production areas of the facility, maintenance, and management. They were specifically asked to review the relative exposure values for production areas. For example, the exposure estimated for the fluoride furnace operator is slightly higher than the helper; this was confirmed to be correct because the helper stood away from the furnace and supplied materials to the perimeter only. The involvement of the group of long-term employees provided added confidence in our derived estimates.

The JEM and TEM were linked through the listing of the tasks in each job taken from the DWA calculation sheets. For jobs never sampled, the association was through the time–activity information developed with the help of long-term employees and, finally, put into DWA format.

For every job title in the JEM, the chemical and physical form of the exposure was listed. Chemical forms included beryl ore, beryllium metal, beryllium fluoride, beryllium hydroxide, and beryllium oxide; physical forms included dust, fume, or mixed (dust and fume). Individuals from the facility were assigned, based on jobs worked, the number of months exposed to three different chemical forms: nonsoluble beryllium compounds (beryllium metal and oxide), soluble beryllium compounds (beryllium fluoride and hydroxide), and mixed chemical forms. Individuals were similarly assigned to the number of months exposed to the three physical forms: dust (beryllium metal, hydroxide, or oxide), fume (beryllium fluoride), and mixed (mixed dust and fume). This allowed us to evaluate any differences in response due to very small particle size (fume) or larger particle size (dust or mixed).

We used chi-square tests to compare the groups (definite or probable disease vs. sensitized vs. no disease) with respect to discrete outcomes. ANOVA was used to compare the groups with respect to continuous outcomes (age, cumulative, mean, and peak exposure levels). For the three disease outcome group comparisons, a screening *p*-value was set at 0.25, below which the pairwise comparisons between groups (definite or probable disease vs. no disease, definite or probable disease vs. sensitized, sensitized vs. no disease) were further investigated. For the discrete outcomes, further chi-square tests were performed on the resulting 2 × *k* tables. For the continuous outcomes, the linear contrasts for these pairwise comparisons were examined in order to control for multiple comparisons. For ease of presentation, we also used two-sample *t*-tests to examine pairwise comparisons of the groups. These parametric tests were followed by the Wilcoxon rank-sum test, a nonparametric test used to ameliorate the effects of violations of the assumptions for the parametric tests (e.g., normal distribution).

We further explored exposure–response relationships with logistic regression analysis after adjustment for potential confounders (smoking, age, other beryllium exposure). In addition to an analysis where only cases with complete information were included, an analysis was carried out after multiple imputations (Rubin 1987) of cumulative and mean exposure values (missing on 65 of 574 individuals).

*p*-Values are presented as calculated. All analyses were performed using SAS statistical software (version 9.1; SAS Institute, Cary, NC). The results of the spirometry testing are not reported in this article.

## Results

A total of 1,351 individuals were identified to have worked at this facility. A summary of the participation rate for this facility is shown in Table 2. Approximately one-fourth (24.4%) of the cohort died before the medical screening began, and another 10.8% could not be located. Among the 875 individuals located, 160 (11.8%) indicated either that they had worked for the company that owned the facility but at a different location, or that they had completed a job application and underwent a pre-employment physical for work at the facility but had either not been hired or had decided not to accept a job at that plant.

Of the remaining 715 former employees, the participation rate was 63.9% (457 of 715) for completion of all components of the medical screening and 91.3% (653 of 715) for completion of the questionnaire only. Five hundred twenty-eight individuals (73.8%) completed at least the blood and chest radiograph component. Reasons members of the cohort gave for not participating included that the individual *a*) had only worked for a short time; *b*) felt he or she was too old and that testing would not matter; *c*) did not have any health problems; *d*) did not want to jeopardize his or her current health insurance, especially with no compensation available (at the time the individual was contacted); and *e*) felt there was no effective treatment for beryllium disease.

Table 3 compares the demographics of medical screening participants with nonparticipants. Medical screening took place from the fall of 1996 through the summer of 2001. Participants were on average the same age as the nonparticipants, the same sex and race, last worked in a more recent year, and worked on the average 3.3 years longer. Participants were mainly male (91%), and almost all white. Seventy percent had ever smoked cigarettes.

Among the 577 individuals that were tested for beryllium, 110 were referred for follow-up testing at the Hospital of the University of Pennsylvania (Table 4). In addition to the 110 referred, 9 individuals from the facility had previously been diagnosed at the University of Pennsylvania with CBD. All 577 individuals, including the 9 previously diagnosed with CBD, were categorized per the criteria in Table 1. The results of this classification are shown in Table 5. Of the cohort, 7.6% (44) had probable or definite CBD, 2.1% (12) had possible CBD, 6.9% (40) were sensitized to beryllium, and 4.0% (23) were possibly sensitized.

Table 6 shows the predictive power of having unrecognized CBD documented by bronchoscopy based on the results of the screening tests performed. Having two positive BeLPTs and scarring on the chest radiograph, involving either all zones or the lower zones only, had the highest predictive value for the development of CBD (100%). In descending order for the other combination of tests, the predictive values for CBD were scarring on the radiograph in all zones with negative BeLPT (75%), positive BeLPT (48.3%), scarring in the upper zones with negative BeLPT (40%), and scarring on the chest radiograph just in the lower zones (7.7%).

There were 33 cases of definite/probable CBD among production workers, 5 among clerical/office workers, 3 in engineers, 1 in a supervisor/inspector, 1 in a laboratory worker, and 1 in an industrial hygiene technician. There were 27 cases of sensitization among production workers, 10 among clerical/office workers, 2 among engineers, and 1 in a nurse.

Table 7 shows the occurrence of definite and probable CBD and sensitization by first decade worked, last decade worked, and duration of years worked. The mean year of first exposure for definite/probable CBD was 1963, for sensitized cases it was 1965, and for the normal group it was 1964. Further, the mean year last exposed for definite/probable, sensitized, and normal individuals was 1973, 1968, and 1971, respectively. The mean duration of exposure for definite/probable, sensitized, and normal individuals was 9.4 years, 2.7 years, and 8.7 years, respectively.

Tables 8–12 show the occurrence of definite and probable CBD and sensitization by the peak, average, and cumulative exposure metric, by chemical and physical form of beryllium and the OSHA (2005) standard of 2 μg/m3 and the DOE (1999) standard of 0.2 μg/m3. Individuals who were sensitized had a lower total cumulative and peak exposure (Table 8), lower nonsoluble cumulative and average exposure (Table 11), and lower dust and mixed exposure (Table 10). Individuals with CBD had a lower soluble (Table 9) and fume exposure (Table 10). The mean beryllium exposure levels for the DWA categories in Table 11 were 0, 1.23, and 8.95 μg/m3, respectively and in Table 12 were 0.14, 1.19, and 4.76 μg/m3, respectively.

## Discussion

The prevalence of CBD and sensitization to beryllium in former workers at this beryllium production facility in eastern Pennsylvania was high: 7.6% with CBD, 6.9% with sensitization, 2.1% with possible CBD, and 4.0% with possible sensitization. This facility operated from 1957 to 1978. Representative exposure estimates for tasks ranged from 0.9 to 84.0 μg/m3 in the 1960s, although most time-weighted averages were below the OSHA (2005) standard of 2 μg/m3, ranging from 1.1 to 2.5 μg/m3. Exposure estimates in the 1970s were lower, with representative tasks ranging from 0.5 to 16.7 μg/m3 and time-weighted averages ranging from 0.7 to 3.5 μg/m3.

The 14.5% prevalence of CBD and sensitization in the cohort we studied contrasts with overall prevalence reports of 3.3% among nuclear workers from Rocky Flats (Stange et al. 2001), 1.8–5.9% from beryllium ceramics manufacturing (Kreiss et al. 1993b, 1996), and 4.6% from a beryllium production facility (Kreiss et al. 1997). Our overall prevalence is similar to the prevalence reports for more highly exposed subgroups from these studies, such as machinists (Kreiss et al. 1996). Our higher overall prevalence rate reflects both the level and the widespread exposure to beryllium in the facility we studied, where 11 definite/ probable cases occurred among nonproduction workers such as clerical, supervisory, and engineering staff and 13 sensitization cases occurred in clerical/office personnel. Our mean and range of cumulative exposure, which was 199.25 μg-year/m3 (0.0–3970.61 μg-year/m3, are appreciably higher than estimates reported in other studies: 6.09 μg-year/m3 (0.15–10.64 μg-year/m3) (Kelleher et al. 2001), 1.35 μg-year/m3 (estimated range, 0–6.41 μg-year/m3) (Viet et al. 2000), and no mean provided (estimated range, 0.9–41.2 μg-year/m3) (Henneberger et al. 2001). An additional factor that probably contributes to the higher prevalence of CBD in our cohort is the long latency since last exposure, which would have allowed a higher proportion of individuals who were sensitized to progress on to CBD than in other cohorts that have been studied (Newman et al. 2005). Most previous prevalence studies of beryllium-exposed workers have been of current employees (Henneberger et al. 2001; Kelleher et al. 2001; Kreiss et al. 1996), or they have included former workers (Stange et al. 2001) but have not presented the results separately for current and former workers. One study similar to ours only had formerly exposed individuals (Kreiss et al. 1993b). This latter study, unlike ours, found no individuals with sensitizations alone without CBD. This would suggest that the higher prevalence of CBD in our study population was not solely related to the long latency since last exposure because we would have expected a lower rate of sensitization alone without CBD if increased prevalence of CBD was solely caused by the long latency.

Despite the fact that there is an overall increase of beryllium disease in working populations with higher exposure to beryllium, investigators have been unable to show a clear-cut exposure response between air concentrations of beryllium and CBD or sensitization (Henneberger et al. 2001; Kelleher et al. 2001; Viet et al. 2000). This has led researchers to examine the possible role of particulate size (Kelleher et al. 2001; McCawley et al. 2001) and skin exposure (Tinkle et al. 2003).

We found no difference in duration of exposure for individuals with CBD versus those who had no evidence of beryllium disease, but we did find that those who were sensitized had begun work later, last worked longer ago, and had a shorter duration of exposure than did those with CBD or those who tested normal (Table 7). This difference for individuals with sensitization was also true for cumulative and peak exposure (Table 8), cumulative mixed and cumulative and mean nonsoluble exposure (Table 9), cumulative and mean dust, and cumulative mixed exposure (Table 10). On the other hand, cumulative and mean soluble and cumulative and mean soluble fume exposures were lower for CBD (Table 10).

In sum, we either found no exposure response or the significant exposure responses we did find were in the opposite direction than expected, with individuals with CBD or sensitization having less estimated exposure than those with no beryllium disease. The risk of CBD compared with sensitization if a person’s mean exposure was below the current DOE (1999) permissible level of 0.2 μg/m3 was less than if their mean level was > 0.2 μg/m3 but below the current OSHA (2005) permissible exposure level of 2 μg/m3 (Table 11). However, only being exposed to beryllium less than either the DOE or the OSHA time-weighted average did not protect a worker from the development of CBD or sensitization. There were only two people in the cohort whose highest level of exposure was never above the 0.2 μg/m3 DOE standard. CBD and sensitization occurred even if the highest level of exposure was never greater than the 2 μg/m3 OSHA standard, and our data would suggest that peak exposure levels > 0.2 μg/m3 were as harmful as even higher peak exposure levels > 2 μg/m3 (Table 12).

A possible explanation for the failure to find an association between increased beryllium disease and sensitization and increased levels of exposure is that this analysis did not consider the role of genetic predisposition to both sensitization and disease. Because the genetic marker glu69 on HLA-DPB1 has been associated with 80–90% of cases of both CBD and sensitization, a better control group for this analysis would be HLA-DPB1 glu69–positive individuals who did not have CBD or sensitization. We have recently been funded to test our population for this marker and thus will eventually be able to determine the interaction of exposure and genetic predisposition.

The finding of higher working lifetime beryllium exposures in those with CBD compared with those who are just sensitized suggests that the body burden of beryllium might relate to the severity of disease in those with a genetic predisposition. Our finding that sensitized individuals compared with individuals with CBD had a higher exposure to beryllium in a soluble form and to fumes of beryllium supports this hypothesis (in this facility soluble beryllium and fume is practically equivalent, r = 0.94). Presumably the soluble forms of beryllium would be more likely to be mobilized and eliminated and result in a lower body burden of beryllium compared with a similar exposure to insoluble beryllium.

Individuals who have recently converted their PPD (purified protein derivative) skin test for tuberculosis to positive may, after treatment, revert to a negative PPD (Tager et al. 1985). Thus, with decreasing or elimination of the antigen, the cellular immune response (i.e., PPD reaction) may fade or be eliminated. Because the PPD reaction is similar to BeLPT, this suggests that a decreased immune response to beryllium may occur in individuals with a lower body burden of beryllium (i.e., antigen). Thus, a reduced immune response to beryllium may account for the association of beryllium sensitization with a lower body burden of insoluble beryllium or predominantly soluble beryllium exposure compared with individuals with CBD. An alternative explanation that less soluble beryllium exposure is confounded by elevated levels of other forms of beryllium is not supported by analyzing potential correlations between levels of exposures to the different forms of beryllium.

Other researchers have suggested the importance of skin exposures to the development of beryllium disease. We have no data to directly address whether skin exposure is of importance in the development of beryllium disease in this cohort. However, others have hypothesized that small particle size increases the likelihood of both inhalation and skin absorption and exposure (McCawley et al. 2001). Our data showed the opposite results with reduced levels of exposure to fume, which would be the smallest particle size form of exposure that occurred in this facility, and CBD (Table 10).

A limitation of our study is the uncertainty in the exposure estimates. The exposure metrics developed for study participants were based on relatively sparse data, with interpolation from measurement data for years when no data were available. Major gaps in the data were associated with the mid-1960s and from 1977 to 1981. Exposure estimates for the earlier time period were based on measurements in preceding or succeeding years; for the later time period, estimates for the mid-1970s were extended into the later years. These decisions were based on plant history and conversations with long-term workers. All interpolation was accomplished using preestablished rules and was independent of any knowledge of disease status. The use of professional judgment like this is often required in retrospective exposure assessment studies. Because the exposure estimates were created for jobs and tasks, without knowledge of a work history or disease status, it is likely that this mis-classification would be nondifferential, attenuating any ability to detect exposure–response relations (Checkoway et al. 1991; Copeland et al. 1977).

A further limitation relates to the effect of nonparticipants on study results. The overall participation rate was high and nonparticipants were generally similar to participants except their duration of exposure was less. However, it is possible that the 11% of the total cohort that did not participate had a lower rate of CBD because asymptomatic individuals might be less motivated to participate. On the other hand, the 24% of the cohort who were deceased at the initiation of our study and the 11% we could not locate might be expected to have a higher prevalence of disease.

A third limitation of our study is that we used a single laboratory for the blood lymphocyte testing for beryllium in a one-time screening. It has recently been reported that the use of a single laboratory results in false negative results of 20–30% (Stange et al. 2004). Because radiographs were part of our screening, we would expect the false negative rate for CBD to be lower than the potential false negative rate for sensitization. Because our cohort was no longer exposed to beryllium, it is less likely that repeat screening will identify additional cases of CBD or sensitization, as has been shown in currently exposed cohorts (Newman et al. 2001).

The participation level of individuals who warranted more extensive testing after the initial screening is another limitation of this study. Only 56 of the 110 (51%) individuals who screened positive by radiograph or BeLPT elected to have a bronchoscopy. Participation in more extensive testing was similar in those with positive radiographs (47%) and those with abnormal positive BeLPT only (57%). The lack of a biopsy and broncholavage in half of the individuals who were positive on the initial medical screening means we may have misclassified individuals into the definite/ probable CBD and sensitization groups. This misclassification would decrease the likelihood of finding an exposure–response or other relationship with CBD or sensitization. To minimize misclassification errors, we excluded cases classified as possible CBD or possible sensitization from both the disease and normal groups during analysis. However, we included four individuals classified as CBD because of a diagnosis at the University of Pennsylvania before our study, although these individuals never had evidence of sensitization in their bronchial lavage fluid or blood. We are not aware of any reason how misclassification could cause the inverse relationship between exposure and disease that we found.

A final limitation is that multiple comparisons were made in Tables 9 and 10. Adjustments for these multiple comparisons can be made by tripling the p-value reported, using the properties of the Bonferroni inequality. If this were done, a number of the associations would no longer be statistically significant in Tables 9 and 10. Given the consistent direction of the findings, our conclusions concerning soluble and nonsoluble forms of beryllium remain unchanged even if this adjustment were made.

In conclusion, this cohort is a high-risk group for the development of CBD and sensitization. The development of beryllium disease has continued to occur years after exposure has ceased. Former beryllium workers and their health care providers need to be aware of this ongoing risk. A combination of two positive BeLPTs and an abnormal chest radiograph on the initial medical screening was the best predictor of the presence of CBD. However, there were individuals who had CBD with an abnormal chest radiograph, involving all or just the upper lobes, and negative BeLPT (Table 6).

We were unable to show an exposure–response relationship. The inclusion of genetic data combined with exposure data may better define which individuals in this cohort are at a particularly high risk of development of CBD and/or sensitization and may account for the absence of the typical exposure–response seen with other environmental or occupational toxins. We are currently performing molecular typing of DRB1 and DPB1 alleles on individuals with CBD and sensitization and a sample of those who tested normal to investigate for a possible gene–exposure relationship.

This cohort is a high-risk group for the development of CBD and sensitization. The development of beryllium disease has continued to occur years after exposure has ceased. Former beryllium workers and their health care providers must be kept aware of this ongoing risk.

The results of this study show that current occupational health standards for beryllium do not provide adequate protection against the development of CBD or sensitization. Twenty-four percent of the workforce that was exposed to beryllium below the current OSHA (2005) allowable threshold limit value developed CBD or sensitization. Similar levels of adverse outcomes (21%) were seen in those exposed to beryllium below the time-weighted average DOE (1999) guideline of 0.2 μg/m3. Even the more protective time-weighted average of 0.02 μg/m3 proposed by the American Conference of Governmental and Industrial Hygienists (2005) did not eliminate adverse outcomes. The identification of cases of CBD and sensitization in this population at levels of cumulative exposure lower than current standards or guidelines underscores the need to more fully understand the determinants of exposure (e.g., peak, physical/chemical form) that may contribute to disease risk, so that these may be included in standard setting.

## Figures and Tables

**Table 1:** Criteria for beryllium disease categories.

Disease category	Bronchial lavage	Biopsy granuloma	Chest radiograph	Blood LPT	Spirometry
CBD	+ BAL LPT	Positive			
	Not done	Positive		Two + LPTs	
	Not done	Positive	Upper lobe fibrosis		
Probable CBD	+ BAL LPT		Upper lobe fibrosis		
	Not done		Upper lobe fibrosis	Two + LPTs	
Possible CBD	Not done		Upper lobe fibrosis	– LPT	
	Not done		Upper lobe fibrosis	Single + LPT and no retest	
Sensitization	– BAL LPT	Negative	Normal	Two + LPTs	Normal
	+ BAL LPT	Negative	Normal		Normal
	Not done		Lower or midlobe fibrosis or normal	Two + LPTs	Normal
Possible sensitization				Single + LPT and no retest or – LPT retests	

Abbreviations: –, negative; +, positive; BAL, bronchoalveolar lavage; LPT, lymphocyte proliferation test.

**Table 2:** Summary of cohort participation.

	No. (%)
Cohort	1,351
Deceased	330 (24.4)
Unable to locate	146 (10.8)
Contacted	875
Denied ever working at facility	160 (11.8)
Potential participants	715
Medical testing	653 (91.3)
Questionnaire only	79
Questionnaire and blood	22
Questionnaire and chest radiograph	6
Questionnaire, chest radiograph, and blood	71
Questionnaire, chest radiograph, and PFTs	12
Questionnaire, PFTs, and blood	6
All components (questionnaire, chest radiograph, blood, and PFTs)	457
Refusals	62 (8.7)

PFT, pulmonary function test.

**Table 3:** Demographics of medical screening participants versus those who completed questionnaire only.^a^

Characteristic	Completed questionnaire only^b^	Medical screening participants^c^
Birth year (mean ± SE)	1936 ± 1.18	1935 ± 0.44
Male sex (%)	91.8	90.8
White race (%)	100.0	99.8
Duration (no.) of years worked (mean ± SE)	5.2 ± 0.85	8.5 ± 0.40*
Last year worked (mean ± SE)	1969 ± 0.89	1971 ± 0.32*

a Sixty-two individuals who refused to complete questionnaire and medical screening are not included.

b Includes 79 individuals who completed only the questionnaire.

c Includes 574 individuals who completed questionnaires and some part of medical screening.

*p < 0.05.

**Table 4:** Reason for referral for follow-up testing.

	No. (%)
Blood	
Two positive BeLPTs	53 (9.2)
Chest radiograph	
Profusion > 1/0 per at least two B readers	50 (8.7)
Blood and chest radiograph^a^	7 (1.2)
Total referred	110^b^ (19.1)

a Met criteria for both blood and chest radiograph referral.

b Nine (1.6%) additional individuals in the cohort met the study’s criteria for CBD based on testing performed before the study’s medical screening.

**Table 5:** Disease categorization of medical test results (n = 577).

Disease category	No. (%)
Definite CBD	32 (5.5)
Probable CBD	12 (2.1)
Possible CBD	12 (2.1)
Sensitized	40 (6.9)
Possibly sensitized	23 (4.7)
No CBD and/or sensitization	458 (79.4)

**Table 6:** Predictive power of having unrecognized CBD documented by bronchoscopy based on results of BeLPT and chest radiograph.

	Total no. having bronchoscopy	Confirmed CBD cases [No. (%)]
Blood		
Two positive BeLPTs	29	14 (48.3)
Chest radiograph	22	6 (27.3)
Profusion > 1/0 at least two B readers		
All zones	4	3 (75.0)
Upper zones only	5	2 (40.0)
Lower zones only	13	1 (7.7)
Blood and chest radiograph^a^	5	5 (100.0)
All zones	3	3 (100.0)
Upper zones only	0	NA
Lower zones only	2	2 (100.0)

NA, not applicable.

a Met criteria for blood and chest radiograph referral.

**Table 7:** Development of definite/probable CBD and sensitization by decade of first and last exposure and duration of exposure.

	Decade of first exposure [No. (%)]			Decade of last exposure [No. (%)]				Duration of exposure (years) [No. (%)]			
Disease outcome	1950s	1960s	1970s	1950s	1960s	1970s	1980s	< 1	1 to < 5	5 to < 15	≥15
Definite/probable CBD	14 (34)	17 (41)	10 (24)	3 (8)	8 (21)	22 (56)	6 (15)	10 (24)	8 (20)	11 (27)	12 (29)
Sensitization	7 (18)	22 (56)	10 (25)	1 (3)	20 (57)	14 (40)	0 (—)	9 (23)	19 (49)	9 (23)	2 (5)
Normal	112 (27)	222 (54)	75 (18)	26 (7)	126 (33)	177 (46)	58 (15)	70 (17)	136 (33)	107 (26)	99 (24)

For the decade of first exposure, p = 0.03 for sensitization vs. normal. For the decade of last exposure, p = 0.03 for sensitization vs. definite/probable and p = 0.008 for sensitization vs. normal. For the duration of exposure, p = 0.008 for definite/probable vs. sensitization and p = 0.03 for sensitization vs. normal.

**Table 8:** Development of definite/probable CBD and sensitization by average cumulative, average mean, and peak exposure (± SE).

Disease outcome	No. of individuals	Mean cumulative exposure (μg-year/m^3^)	Mean exposure (days)	Mean average exposure (μg/m^3^)	Mean peak exposure (μg/m^3^)
Definite/probable CBD	40	181 ± 29	3,483 ± 550	8.7 ± 0.8	81 ± 14
Sensitization	37	100^a^ ± 23	1,934^b^ ± 55	7.1 ± 0.9	53^c^ ± 14
Normal	377	209 ± 16	3,359 ± 176	8.3 ± 0.3	87 ± 13

a p = 0. 03 for sensitization vs. definite/probable, and p = 0. 0003 for sensitization vs. normal.

b p = 0.047 for sensitization vs. definite/probable, and p = 0.02 for sensitization vs. normal.

c p = 0. 01 for sensitization vs. normal.

**Table 9:** Development of definite/probable CBD and sensitization by chemical form of beryllium, mixed, nonsoluble, and soluble: mean cumulative, mean average, and mean peak exposure levels.

		Mixed			Nonsoluble			Soluble		
Disease outcome	No.	Cumulative (μg-year/m^3^)	Mean (μg/m^3^)	Peak (μg/m^3^)	Cumulative (μg-year/m^3^)	Mean (μg/m^3^)	Peak (μg/m^3^)	Cumulative (μg-year/m^3^)	Mean (μg/m^3^)	Peak (μg/m^3^)
Definite/probable CBD	40	50	3.7	2.1	126	7.6	4.6	5.8^a^	0.8^b^	2.1
Sensitization	37	20^c^	2.3	4.4	61^d^	5.4^e^	2.8	19	2.3	4.4
Normal	377	49	3.4	3.5	128	7.4	4.5	26	1.6	3.6

a p < 0.0001 for definite/probable vs. normal.

b p = 0.02 for definite/probable vs. normal.

c p = 0.0005 for sensitization vs. normal.

d p = 0.04 for sensitization vs. definite/probable, and p = 0.003 for sensitization vs. normal.

e p = 0.02 for sensitization vs. normal.

**Table 10:** Development of definite/probable CBD and sensitization by physical form of beryllium, dust, fume, and mixed: mean cumulative, mean average, and mean peak exposure levels.

		Dust			Fume			Mixed		
Disease outcome	No.	Cumulative (μg-year/m^3^)	Mean (μg/m^3^)	Peak (μg/m^3^)	Cumulative (μg-year/m^3^)	Mean (μg/m^3^)	Peak (μg/m^3^)	Cumulative (μg-year/m^3^)	Mean (μg/m^3^)	Peak (μg/m^3^)
Definite/probable CBD	40	128	7.4	4.6	4^a^	0.7^b^	0.3	49	3.6	2.1
Sensitization	37	66^c^	5.1^d^	3.5	17	2.3	3.1	17^e^	2.4	4.4
Normal	377	138	7.1	5.4	20	1.4	1.3	46	3.3	3.5

a p = 0.0002 for definite/probable vs. normal.

b p = 0.03 for definite/probable vs. normal.

c p = 0.0021 for sensitization vs. normal.

d p = 0.009 for sensitization vs. normal.

e p = 0.0004 for sensitization vs. normal.

**Table 11:** Development of definite/probable CBD and sensitization by the American Conference of Governmental and Industrial Hygienists notice of intended change, current OSHA, and DOE DWA threshold levels.

	Mean DWA exposure (μg/m3) [n (%)]			
Disease outcome	0 to < 0.02	0.02 to < 0.2	0.2 to < 2	≥2
Definite/probable CBD	1 (7)	0 (0)	4 (17)	35 (8)
Sensitization	2 (14)	0 (0)	2 (8)	33 (8)
Normal	11 (79)	0 (0)	18 (75)	348 (84)

**Table 12:** Development of definite/probable CBD and sensitization by highest exposure.

	Highest exposure level (μg/m^3^) [n (%)]		
Disease outcome	0 to < 0.2	0.2 to < 2	≥2
Definite/probable CBD	0 (—)	18 (9.3)	22 (8.5)
Sensitization	0 (—)	19 (9.8)	18 (7.0)
Normal	2 (100)	157 (80.9)	218 (84.5)
