# Erratum

**Published:** 2006-04

In the article by Rosenman et al. [ Environ Health Perspect 113:1366–1372 (2005)], the authors noted errors in Tables 8–11, which resulted from a programming error. Because of this error, the overall mean daily weighted averages (DWAs) were greater than they should have been. The corrected tables are shown below.

The correct mean exposure levels for the DWA categories in Table 11 were 0.13, 1.15, and 3.13 μg/m^3^.

## Figures and Tables

**Table 8:** Development of definite/probable CBD and sensitization by average cumulative, average mean, and peak exposure (± SE).

Disease outcome	No. of individuals	Mean cumulative exposure (μg-year/m^3^)	Mean exposure (days)	Mean average exposure (mg/m^3^)	Mean peak exposure (mg/m^3^)
Definite/Probable CBD	40	181 ± 29	3483 ±50	1.6 ± 0.21	3.1 ± 0.36
Sensitization	37	100 ± 23^a^	1934 ±5^b^	1.6 ± 0.16	2.9 ± 0.4
Normal	377	209 ± 16	3359 ±76	1.6 ± 0.06	3.3 ± 0.13

^a^*p* = 0.03 for sensitization vs. definite/probable and *p* = 0.0003 for sensitization vs. normal.

^b^*p* = 0.047 for sensitization vs. definite/probable and *p* = 0.02 for sensitization vs. normal.

**Table 9:** Development of definite/probable CBD and sensitization by chemical form of beryllium, mixed, nonsoluble, and soluble: mean cumulative, mean average, and mean peak exposure levels.

		Mixed			Nonsoluble			Soluble		
Disease outcome	No.	Cumulative (μg-year/m^3^)	Mean (μg/m^3^)	Peak (μg/m^3^)	Cumulative (μg-year/m^3^)	Mean (μg/m^3^)	Peak (μg/m^3^)	Cumulative (μg-year/m^3^)	Mean (μg/m^3^)	Peak (μg/m^3^)
Definite/probable CBD	40	50	0.9	3.7	126	1.7	5.1	5.8^a^	0.9	1.1
Sensitization	37	20^b^	0.8	6.8	61^c^	1.6	5.1	19	0.8	3.3
Normal	377	49	0.9	5.9	128	1.6	6.1	26	0.9	2.7

^a^*p* < 0.0001 for definite/probable vs. normal.

^b^*p* = 0.0005 for sensitization vs. normal.

^c^*p* = 0.04 for sensitization vs. definite/probable, and *p* = 0.003 for sensitization vs. normal.

**Table 10:** Development of definite/probable CBD and sensitization by physical form of beryllium, dust, fume, and mixed: mean cumulative, mean average, and mean peak exposure levels.

		Dust			Fume			Mixed		
Disease outcome	No.	Cumulative (μg-year/m^3^)	Mean (μg/m^3^)	Peak (μg/m^3^)	Cumulative (μg-year/m^3^)	Mean (μg/m^3^)	Peak (μg/m^3^)	Cumulative (μg-year/m^3^)	Mean (μg/m^3^)	Peak (μg/m^3^)
Definite/probable CBD	40	128	1.6	5.2	4^a^	0.5	0.8^b^	49	0.9	3.7
Sensitization	37	66^c^	1.5	5.1	17	0.9	3.3	17^d^	0.8	6.8
Normal	377	138	1.5	6.6	20	0.6	1.9	46	0.9	5.9

^a^*p* = 0.0002 for definite/probable vs. normal.

^b^*p* = 0.04 for definite/probable vs. normal.

^c^*p* = 0.0021 for sensitization vs. normal.

^d^*p* = 0.0004 for sensitization vs. normal.

**Table 11:** Development of definite/probable CBD and sensitization by the American Conference of Governmental and Industrial Hygienists notice of intended change, current OSHA, and DOE DWA threshold levels.

	Mean DWA exposure (μg/m^3^) [*n* (%)]			
Disease outcome	0 to < 0.02	0.02 to < 0.2	0.2 to < 2	≥ 2
Definite/probable CBD	0 (0)	0 (0)	33 (10)	7 (6)
Sensitization	0 (0)	0 (0)	24 (7)	13 (11)
Normal	0 (0)	3 (100)	279 (83)	95 (83)

